# A review of the genus *Megapulvinaria* Young (Hemiptera, Coccoidea, Coccidae) from China, with a description of a new species

**DOI:** 10.3897/zookeys.228.3211

**Published:** 2012-10-16

**Authors:** Fang Wang, Ji-Nian Feng

**Affiliations:** 1 Key Laboratory of Plant Protection Resources and Pest Management, Ministry of Education, Entomological Museum, College of Plant Protection, Northwest A & F University, Yangling, Shaanxi Province, 712100, China

**Keywords:** Hemiptera, Coccoidea, soft scale, taxonomy, China

## Abstract

Prior to this study, only *Megapulvinaria maxima* (Green) was known from China. However, a new species *Megapulvinaria beihaiensis* Wang & Feng, **sp. n.** is described below and *Megapulvinaria maxima* is redescribed. A key is provided for the five species now placed in this genus.

## Introduction

Soft scale or Coccidae is the third largest family after Diaspididae and Pseudococcidae within the superfamily Coccoidea (Ben-Dov 2012). Soft scale insects have a world-wide distribution and many of them are important pests on agricultural and horticultural crops and amenity plantings (Henderson and Hodgson 2005). China has a fauna of soft scale insects with a total of at least 125 species belonging to 46 genera (Tang 1991, Tao 1999, Wu 1999, Martin and Lau 2011). Some species that occur in China, such as *Ceroplastes rubens* and *Ceroplastes japonicus*, can cause deformation or death of plants shoots and lead to great economic losses due to their feeding. However, some species present in China can be considered beneficial, such as *Ericerus pela*, whose wax provides an important raw material for many industries (Tang 1991).

The genus *Megapulvinaria* was erected by Young (1982), with *Pulvinaria maxima* Green, 1904 as its type species, and belongs to the tribe Pulvinariini, subfamily Coccinae. Three more species *Megapulvinaria burkilli* (Green, 1908), *Megapulvinaria orientalis* (Reyne, 1963) and *Megapulvinaria maskelli* (Olliff, 1891) have been subsequently added (Avasthi and Shafee 1991, Ben-Dov 1993, Hodgson 1994).

Previously, only *Megapulvinaria maxima* was known from China but a new species has now been discovered. The adult female of *Megapulvinaria maxima* is redescribed, the adult female of the new species *Megapulvinaria beihaiensis* Wang & Feng sp. n. is described and a key is provided for separation of the five species now known in this genus.

## Materials and methods

Specimens were slide mounted using the method recommended by Hodgson and Henderson (2000). The morphological terminology of the mounted specimens used in the descriptions mainly follows Hodgson (1994). Characters were examined under a Nikon microscope. Illustrations were drawn from mounted adult female specimens, with the dorsum depicted on the left side and the venter on the right side, and with enlargements of important characters shown around the main illustration. All measurements were given in micrometers (µm) or millimeters (mm).

All specimens are deposited in the Entomological Museum of Northwest A & F University, Yangling, Shaanxi, China (NWAFU).

### Checklist of known species of the genus *Megapulvinaria* Young

*Megapulvinaria maxima* (Green, 1904); China (Guangxi, Yunnan, Taiwan), Thailand, India, Indonesia, Philippines, Sri Lanka, Vietnam, Papua New Guinea, Chuuk Islands.

*Megapulvinaria burkilli* (Green, 1908); India.

*Megapulvinaria orientalis* (Reyne, 1963); Thailand.

*Megapulvinaria maskelli* (Olliff, 1891); Australia.

*Megapulvinaria beihaiensis* sp. n.; China (Guangxi).

## Taxonomy

### Genus Megapulvinaria

Young, 1982

http://species-id.net/wiki/Megapulvinaria

Megapulvinaria Young, 1982: 162. Type species: *Pulvinaria maxima* Green, 1904. By original designation and monotype.

#### Generic diagnosis.

**Adult female.** Body elongate oval to broad oval; stigmatic clefts distinct. **Dorsum.** Dorsal setae spinose or conical. Dorsal submarginal tubercles absent. Preopercular pores present or absent. Dorsal tubular ducts present or absent. Eyespots generally displaced onto dorsum (marginal on *Megapulvinaria maxima*). Anal plates together quadrate, each plate with 2 spinose and/or truncate setae along inner margin, a similar seta on apex and a spinose seta present in discal position (possibly on outer margin of *Megapulvinaria maskelli*). Anal ring with 6 setae. **Margin.** Marginal setae stout, apex truncate or bidentate, and with 2 types present, one shorter and broader than other (about same length and one slightly broader than other both in *Megapulvinaria maskelli* and *Megapulvinaria beihaiensis*); broader setae on head and posterior margins of abdomen (0–3 broader setae present between two stigmatic clefts in *Megapulvinaria beihaiensis*). Stigmatic clefts deep or shallow, each with 3–12 stigmatic spines. **Venter.** Antennae 7–9 (mostly 8) segmented. Legs well-developed, each with a tibio-tarsal articulation and an articulatory sclerosis, each claw with a denticle on the widest part. Pregenital setae 2 pairs. Spiracular disc-pores each mainly with 5 loculi. Pregenital disc-pores each mainly with 10 loculi, restricted to abdominal segments. Ventral tubular ducts of three types, with a submarginal band of small tubular ducts; median area of head, thorax, and anterior 1–3 abdominal segments with large ducts each with both outer and inner ductules broad or stout (anterior submargin and all median area in *Megapulvinaria maskelli*); posterior abdominal segments of moderately tubular ducts.

#### Distribution.

Oriental and Australian regions.

#### Key to all adult females of *Megapulvinaria*

**Table:** 

1	Dermal areolations absent	*Megapulvinaria burkilli* (Green)
–	Dermal areolations present	2
2	Anal plates with dorsal reticulations	3
–	Anal plates without dorsal reticulations	4
3	With only 3 stigmatic spines in each stigmatic cleft	*Megapulvinaria maskelli* (Olliff)
–	With more than 3 stigmatic spines in each stigmatic cleft	*Megapulvinaria beihaiensis* sp. n.
4	With only 1 pair of interantennal setae present; all of lateral stigmatic spines about same length	*Megapulvinaria orientalis* (Reyne)
–	With 2–5 pairs of interantennal setae present; not all of lateral stigmatic spines about same length	*Megapulvinaria maxima* (Green)

### Megapulvinaria maxima

(Green, 1904)

http://species-id.net/wiki/Megapulvinaria_maxima

Figure 1

Pulvinaria maxima Green 1904: 206.
Pulvinaria thespesiae Green 1909: 259. Syn. by Takahashi 1935: 10.
Eriochiton formosae Takahashi 1929: 64. Syn. by Takahashi 1935: 10.
Megapulvinaria maxima (Green), Young 1982: 162.

#### Material examined.

5 adult females, CHINA, Yunnan, Jingdong, 18. x. 1976 on Pigeonpea (*Cajanus cajan* (L.) Millsp., Leguminosae), Xiao-Ze Chen (NWAFU).

#### Note.

The measurements are based on all 5 specimens.

#### Diagnosis. 

**Adult female. Mounted material.** Body elongate oval, about 4.2–6.2 mm long and 2.7–3.8 mm wide. Anal cleft approximately 1/7 of the body length. Stigmatic clefts deep.

**Dorsum.** Derm membranous. Dermal areolations well developed, each with 1 or 2 dorsal microducts. Dorsal setae conical, with a well-developed basal socket, each 8–16 µm long, scattered throughout. Dorsal simple pores each with a slightly sclerotized margin, randomly distributed. Dorsal microducts each with a very short outer ductule and a longer, fairly broad inner filamentous ductule, sparsely located in dorsal areaolations. Dorsal tubular ducts each with a short outer ductule and a fine inner ductule with a minute terminal gland, sparsely distributed. Preopercular pores absent. Anal plates together quadrate; posterior margin slightly longer than anterior margin, outer angle slightly obtuse; each plate with a large cylindrical seta in discal position, each 34–50 µm long, a large spatulate seta apically, each 52–64 µm long, and with 2 spinose and/or spatulate setae along posterior 1/3rd of inner margin, each 40–56 µm long. Ano-genital fold with 1 pair of long setae and 1 pair of short setae along anterior margin and 2 or 3 pairs lateral margin. Anal ring subcircular, with 2 or 3 rows of translucent pores and 6 anal ring setae. Eyespots present some way onto dorsum, each 80–96 µm wide.

**Margin.**Marginal setae of 2 types: 1) large and stout setae, each 17–38 µm long, with nearly parallel sides, and with either a truncate or a bifid apex, all with well-developed basal sockets, each socket with 1 or 2 small pores; with 96–110 setae between anterior clefts, 36–46 setae on each side between stigmatic clefts, and 84–98 setae between each posterior stigmatic cleft and anal cleft; and 2) quite broad and short setae, each 14–24 µm long, with parallel sides and a truncate, flattened apex, and with a larger basal socket about twice as broad as that of type 1), each socket with 3–8 small pores; latter type of marginal setae only distributed on anterior and posterior ends, with 16–22 setae anteriorly on head and prothorax, 5–12 setae on either side of abdomen near anal cleft. Stigmatic clefts deep; stigmatic spines bluntly spinose and mostly straight, with 4–8 spines in each anterior cleft and 5–10 in each posterior cleft; length of each 42–96 µm, with median 1–3 spines much longer than the lateral spines.

**Venter.** Derm membranous. Antennae 8 segmented, each 505–586 µm long; third segment longest; with 2 pairs of long setae and 1–3 pairs of short interantennal setae. Clypeolabral shield 198–232 µm long, 205–240 µm wide; labium 90–106 µm long, 113–144 µm wide. Legs well-developed, each with a tibio-tarsal articulation and articulatory sclerosis; claws with a denticle on widest part, claw digitules broad and expanded apically, tarsal digitules slender, knobbed and longer than claw digitules; trochanter+femur 239–405 µm, tibia 180–245 µm and tarsus 96–122 µm. With 2 pairs of long pregenital setae present in both segments VI & VII; submarginal setae present in a single row; other setae slender, each 4–10 µm long, quite sparsely distributed. Spiracles normal, spiracular disc-pores each with 5 loculi, present in a broad band between stigmatic cleft and each spiracle. Pregenital disc-pores each mainly with 10 loculi, present around the vulva and on posterior 4 abdominal segments. Ventral microducts scattered. Ventral tubular ducts of 3 types present: 1) a duct with a short outer ductule and a fine inner filament, with a minute terminal gland, present in a complete submarginal band; 2) a duct with outer and inner ductules both broad and with a well-developed terminal gland, present medially on head, thorax and anterior 1 or 2 abdominal segments; and 3) a duct with a moderately long outer ductule and a thin inner ductule slightly longer than outer ductule, with a flower-shaped terminal gland, present medially on posterior abdominal segments and extending and mingling with marginal band of type 1) ducts.

**Figure 1.**
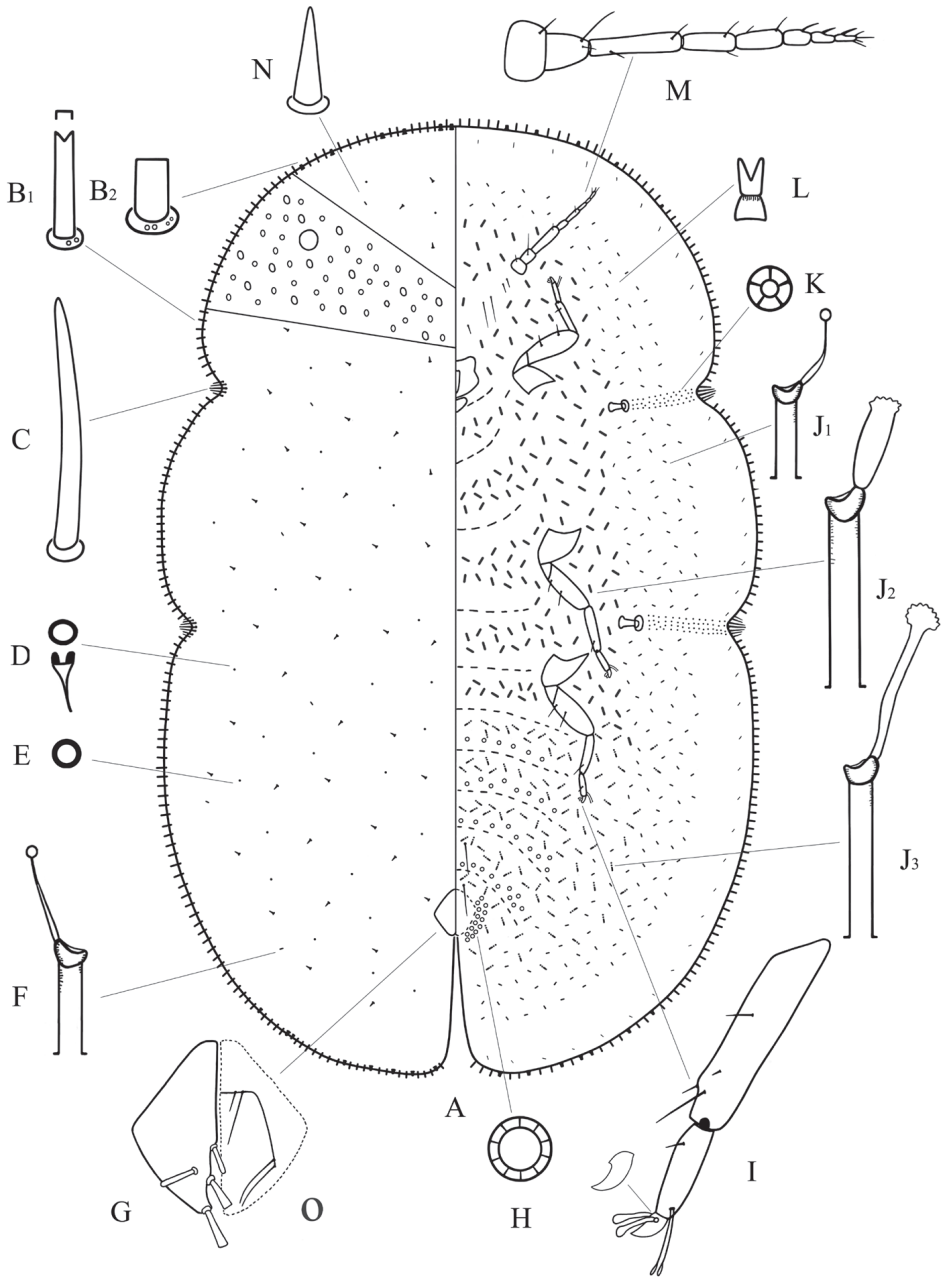
Adult female of *Megapulvinaria maxima* Green, **A** body derm **B1, B2** two kinds of marginal setae **C** stigmatic spine **D** dorsal microduct **E** dorsal pore **F** dorsal tubular duct **G** anal plates **O** ano-genital fold **H** pregenital disc-pore **I** tibio-tarsus of hind leg **J1, J2, J3** ventral tubular ducts **K** spiracle disc-pore **L** ventral microduct **M** antenna **N** dorsal seta.

#### Distribution.

China (Guangxi, Yunnan, Taiwan), Thailand, India, Indonesia, Philippines, Sri Lanka, Vietnam, Papua New Guinea, Chuuk Islands.

#### Comments.

Since Green (1904) originally described *Megapulvinaria maxima*, it had been described and illustrated by many authors, such as Green (1904, 1909), Takahashi (1929), Tang (1991), Hodgson (1994). Our observations agree well with these in descriptions in most respects. Tang (1991) and Hodgson (1994) pointed out the presence of dorsal areolations and denticles on widest part of claws, which Green (1909) and Takahashi (1929) failed to observe respectively. The outer angles of anal plates were obtuse or broadly rounded rather than at a right-angle, as shown by Hodgson (1994). We have confirmed the occurrence of dorsal areolations and denticles, and the outer angles are slightly obtuse in our examined specimens. Moreover, Hodgson (1994) described four types of dorsal pores, but we just observed just two types.

This species is close to *Megapulvinaria burkilli* (Green) (data from Green, 1908), but it can be distinguished from the latter by the following features (character states of *Megapulvinaria burkilli* in brackets): (1) the much larger body size in comparison to the latter (4 mm long, 2 mm wide); and (2) with well-developed dermal areolations present (absent).

### Megapulvinaria beihaiensis

Wang & Feng sp. n.

urn:lsid:zoobank.org:act:56E4CA5F-6C56-431C-AD2B-6A54776BC16B

http://species-id.net/wiki/Megapulvinaria_beihaiensis

Figure 2

#### Material examined.

**Holotype:** adult female. CHINA, Guangxi, Beihai, Haibin Park. 26. vii. 2010, on *Cinnamomum* sp., (Lauraceae), Bin Zhang (NWAFU)

#### Paratypes.

3 adult females, the data same as holotype.

#### Note.

The measurements are based on all 4 specimens.

**Description.** **Adult female.** **Unmounted material.** Adult female yellowish brown or dark brown, elongate oval and with a longitudinal dorsal ridge in dorsal straight median area (materials examined were all immersed in 75% ethanol, and the ovisac was not seen). The specimens collected on the lamina of the host plant.

**Mounted material.** Body elongate oval, about 2.1–3.2 mm long, 1.3–1.7 mm wide. Anal cleft approximately 1/8 of the body length. Stigmatic clefts deep.

**Dorsum.** Derm membranous. Dermal areolations well-developed, each with a dorsal microduct. Dorsal setae conical, with a well-developed basal socket, each 6–11 µm long, scattered throughout. Dorsal simple pores each with a slightly sclerotized margin, randomly distributed. Dorsal microducts each with a very short outer ductule and a long, fairly broad inner filamentous ductule, sparsely located in each dorsal areaolation. Dorsal tubular ducts each with a short outer ductule and a fine inner ductule with a minute terminal gland, sparsely distributed. Preopercular pores absent. Anal plates together quadrate, dorsal surface with reticulations on anterior two-thirds; posterior margin subequal to or slightly longer than anterior margin, outer angle a right-angle; each plate with a blunt spinose seta in discal position, each 34–42 µm long, a large spinose or spatulate seta apically, each 48–54 µm long, and with 2 spinose setae along posterior 1/3rd of the inner margin, each 32–44 µm long, length of plates 146–167 µm, width of single plate 74–88 µm. Ano-genital fold with 1 pair of long setae and 1 pair of short setae along anterior margin and 2 or 3 pairs lateral margin. Anal ring subcircular, with 2 or 3 rows of translucent pores and 6 anal ring setae. Eyespots present some way onto dorsum, each 42–60 µm wide.

**Margin.** Marginal setae of 2 types: 1) stout setae, each 18–30 µm long; each seta with nearly parallel sides and with either a truncate or a bifid apex, all with well-developed basal sockets, each socket with 1 or 2 small pores; with 101–111 setae between anterior clefts, 34–42 setae on each side between stigmatic clefts, and 74–85 setae between each posterior stigmatic cleft and anal cleft; 2) quite strong setae, subequal in length with type 1) but slightly broader; each seta with parallel sides, with a truncate and flattened apex, and with a large basal socket about twice as broad as that of type 1), each socket with 2–8 small pores; with 10–16 setae anteriorly on head and prothorax, 0–3 setae between stigmatic clefts, and 4–10 setae on either side of abdomen near anal cleft. Stigmatic clefts deep; stigmatic spines bluntly spinose and mainly curved apically, with 4 or 5 spines in each anterior cleft and 5–8 spines in each posterior cleft; length of each 34–62 µm, and the median 1–3 spines longer than the lateral spines.

**Venter.** Derm membranous. Antennae 8 segmented, each 346–378 µm long, the third segment longest; with 2 pairs of long setae and 2 or 3 pairs of short interantennal setae. Clypeolabral shield 138–160 µm long, 160–172 µm wide; labium 96–112 µm long, 84–112 µm wide. Legs well-developed, each with a tibio-tarsal articulation and articulatory sclerosis; claws with a denticle on widest part, claw digitules both broad and expanded apically; tarsal digitules slender, knobbed and longer than claw digitules; trochanter+femur 212–245 µm, tibia 136–188 µm and tarsus 54–75 µm. With 2 pairs of long pregenital setae present in both segments VI & VII; submarginal setae present in a single row; other setae slender, 6–20 µm long, quite sparsely distributed. Spiracles normal; spiracular disc-pores each mainly with 5 loculi, present in a broad band between stigmatic cleft and each spiracle. Pregenital disc-pores each mainly with 10 loculi, present around the vulva and on posterior 5 abdominal segments but becoming progressively less frequent anteriorly. Ventral microducts scattered. Ventral tubular ducts of 3 types present: 1) a duct with a short outer ductule and a fine inner filament with a minute terminal gland, present in a complete submarginal band; 2) a duct with a broad outer ductule, a stout inner ductule (as broad as outer ductule in some specimens) and with a well-developed terminal gland, present medially on thorax and anterior abdominal segments; and 3) a duct with a moderately long outer ductule, a thin inner ductule slightly longer than outer ductule, with a flower-shaped terminal gland, present medially on posterior abdominal segments and extending and mingling with marginal band of type 1) ducts.

**Figure 2.**
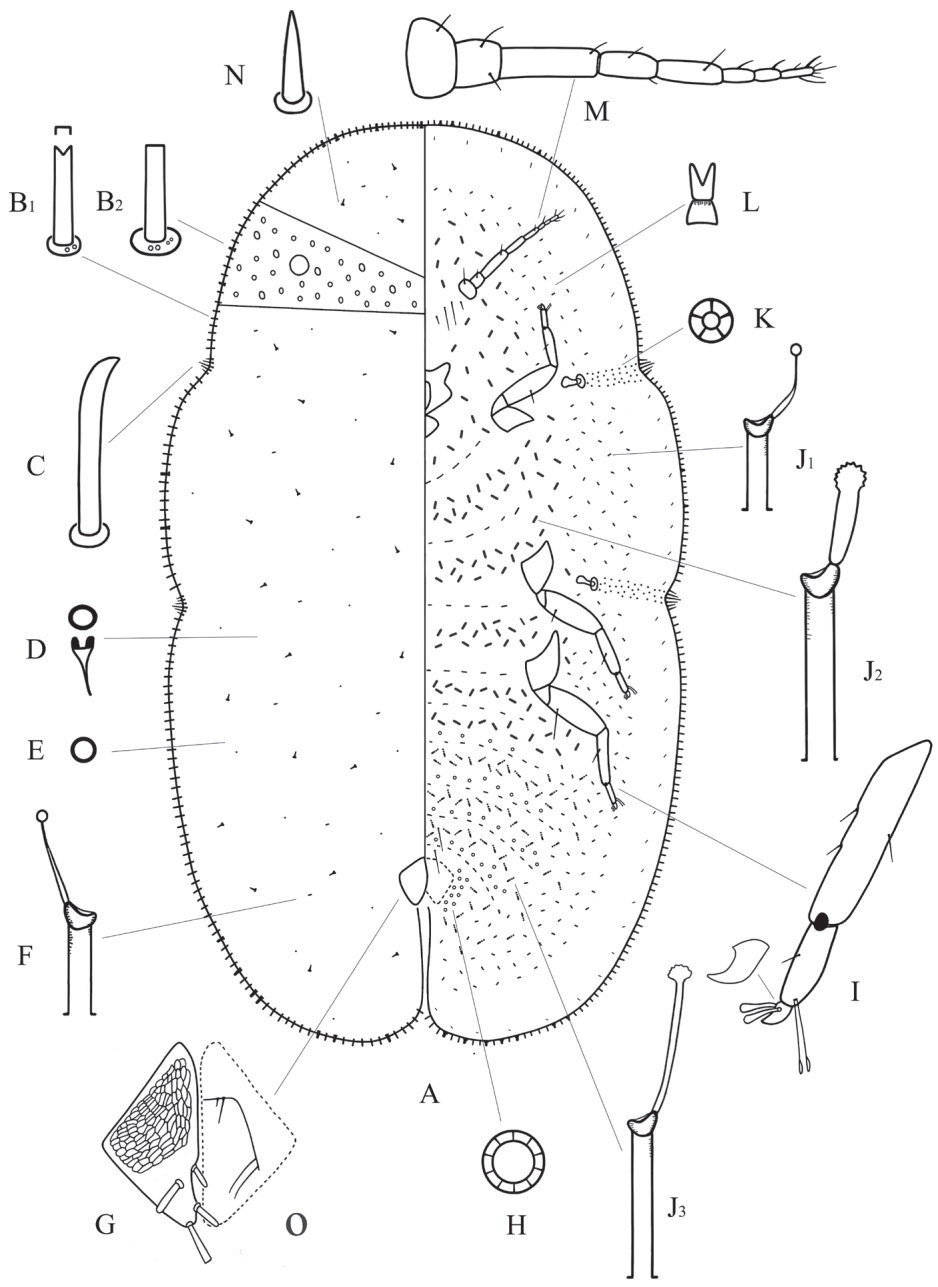
Adult female of *Megapulvinaria beihaiensis* sp. n., **A** body derm **B1, B2** two kinds of marginal setae **C** stigmatic spine **D** dorsal microduct **E** dorsal pore **F** dorsal tubular duct **G** anal plates **O** ano-genital fold **H** pregenital disc-pore **I** tibio-tarsus of hind leg **J1, J2, J3** ventral tubular ducts **K** spiracle disc-pore **L** ventral microduct **M** antenna **N** dorsal seta.

#### Distribution.

China (Guangxi).

#### Etymology. 

The specific epithet is taken from the type locality Beihai.

#### Comments.

This new species resembles *Megapulvinaria maskelli* (Olliff) (data from Qin and Gullan 1992) in having: (1) dorsal reticulations on the anal plates, and (2) 2 types of marginal setae of about same length. However, *Megapulvinaria beihaiensis* can be distinguished by following features (character states of *Megapulvinaria maskelli* in brackets): (1) more than 3 stigmatic spines in each stigmatic cleft (only 3); (2) having dorsal tubular ducts (absent); (3) lacking preopercular pores (present); (4) eyespots displaced onto the dorsum (on the margin); and (5) the marginal setae of much broader basal socket often present between stigmatic clefts (absent).

*Megapulvinaria maskelli*, currently only known from the Australian region, is the only non-Oriental species in this genus and has some distinctive characteristics within *Megapulvinaria*. It differs from other species in having: (1) only 3 stigmatic spines in each stigmatic cleft; (2) eyespots located on margin; and (3) the discal setae possibly on outer margin of anal plates.

## Supplementary Material

XML Treatment for Megapulvinaria

XML Treatment for Megapulvinaria maxima

XML Treatment for Megapulvinaria beihaiensis
